# N-Acetylcysteine in Endometriosis: A Systematic Review of Biological Rationale and Clinical Evidence

**DOI:** 10.3390/antiox15070880

**Published:** 2026-07-16

**Authors:** Rafał Watrowski, Stoyan Kostov, Roberto Tozzi, Tomáš Kupec, Sebastian D. Schäfer, Pierluigi Chieppa, Angel Yordanov, Radmila Sparić, Ingolf Juhasz-Böss, Salvatore Giovanni Vitale, Ibrahim Alkatout

**Affiliations:** 1 Department of Gynecology, Helios Hospital Müllheim, Heliosweg 1, 79379 Müllheim, Germany; 2 Faculty of Medicine, University of Freiburg, 79106 Freiburg, Germany; 3 Department of Gynecology, Hospital “Saint Anna”, 9002 Varna, Bulgaria; drstoqn.kostov@gmail.com; 4 Research Institute, Medical University Pleven, 5800 Pleven, Bulgaria; 5 Unit of Gynecology and Obstetrics, Department of Women and Children’s Health, University of Padua, 35122 Padua, Italy; roberto.tozzi@unipd.it; 6 Department of Gynecology and Obstetrics, University Hospital of the RWTH Aachen, Pauwelsstrasse 30, 52074 Aachen, Germany; tkupec@ukaachen.de; 7 Department of Gynecology and Obstetrics, Clemenshospital Münster, 48153 Münster, Germany; seb.schaefer@alexianer.de; 8 Department of Surgical Sciences, Gynecology and Obstetrics 1, A.O.U. City of Health and Science of Turin, S. Anna Hospital, 10126Turin, Italy; pchieppa@libero.it; 9 Department of Gynecologic Oncology, Medical University Pleven, 5800 Pleven, Bulgaria; angel.jordanov@gmail.com; 10 Faculty of Medicine, University of Belgrade, Dr Subotića 8, 11000 Belgrade, Serbia; radmila@rcub.bg.ac.rs; 11 Clinic for Gynecology and Obstetrics, University Clinical Centre of Serbia, Dr Koste Todorovića 26, 11000 Belgrade, Serbia; 12 Department of Obstetrics and Gynecology, Medical Center—University Hospital Freiburg, 79106 Freiburg, Germany; ingolf.juhasz-boess@uniklinik-freiburg.de; 13 Obstetrics and Gynecology Unit, “Gaspare Rodolico” University Hospital, Department of General Surgery and Medical Surgical Specialties, University of Catania, 95124 Catania, Italy; salvatoreg.vitale@unict.it; 14 Department of Obstetrics and Gynecology, Medical University of Innsbruck, 6020 Innsbruck, Austria; ibrahim.alkatout@i-med.ac.at

**Keywords:** endometriosis, endometrioma, N-acetylcysteine, N-acetyl-L-cysteine, antioxidants, oxidative stress, inflammation, non-hormonal therapy, drug repurposing

## Abstract

Background: Endometriosis is a chronic inflammatory and estrogen-dependent disease in which oxidative stress, immune dysregulation, and lesion-supportive microenvironmental signaling contribute to lesion development. N-acetylcysteine (NAC), a glutathione-replenishing and redox-modulating agent, has been studied as a possible non-hormonal intervention. Methods: Following preregistration in the Open Science Framework (OSF), we performed a PRISMA-compliant systematic review of studies evaluating NAC in endometriosis-related clinical, in vivo, ex vivo, or in vitro settings. We searched PubMed/MEDLINE, EBSCOhost, and the Bielefeld Academic Search Engine (BASE) from inception to 24 May 2026. Risk of bias was assessed with design-specific instruments. Results: Twenty-two studies were included. Clinical evidence comprised six studies and suggested that the findings vary by clinical context. In non-postoperative settings, NAC was associated with reduced pain, decreased NSAID use, and endometrioma shrinkage or stabilization. In contrast, the only postoperative randomized comparison found no added benefit of NAC over continuous oral contraceptive therapy for recurrence or pain. Fertility-related clinical findings were suggestive but preliminary. Across nonclinical models, NAC reduced proliferation, lesion burden, oxidative stress, inflammatory mediators, and, in selected systems, migration-related behavior. Mechanistic studies linked NAC to modulation of ROS-dependent proliferation, TNF-α, COX-2, MMP-2/-9, autophagy-related signaling, oxidation-sensitive nociception, and partial protection of oocyte and embryo competence under endometriosis-associated oxidative conditions. Conclusions: NAC shows biological plausibility and preclinical activity in endometriosis, but clinical evidence is heterogeneous and methodologically limited.

## 1. Introduction

Endometriosis is a chronic gynecologic condition whose systemic effects reach far beyond the visible ectopic lesions [1,2]. The disorder affects at least one in ten women of reproductive age, often begins early in life, and causes a cumulative burden of pain, subfertility, fatigue, reduced work capacity, psychosocial distress, and sustained healthcare utilization [1,2,3]. Endometriosis is diagnosed in 15–71% of women presenting with chronic pelvic pain, and in 9–68% of women presenting with infertility [4]. The interval from symptom onset to diagnosis commonly extends over several years and, in German-speaking cohorts, has approached a decade, with both medical and psychosocial factors contributing to the delay [5,6]. Current guideline recommendations have shifted from mandatory histological confirmation toward symptom- and imaging-based diagnosis [6,7,8]. The Cochrane review on imaging for endometriosis identified promising diagnostic performance for transvaginal sonography and magnetic resonance imaging (MRI) in selected phenotypes and anatomical sites, yet it did not support a simple universal replacement of surgery across the full disease spectrum [9]. Although high-level transvaginal sonography can be remarkably informative in experienced hands, its performance is skill-dependent, anatomically uneven, and less suited to subtle superficial peritoneal disease than to ovarian or deep infiltrating phenotypes [10]. In this context, the research on reliable and non-invasive endometriosis markers detectable in serum [11,12], urine [13], or menstrual effluent [14] is growing. A non-invasive saliva test based on endometriosis-associated miRNA signatures has already reached the market [15,16]; however, its integration into routine diagnostic pathways remains limited [17].

Pathogenetically, endometriosis is a multifactorial disorder in which neither the classical theory of retrograde menstruation, proposed a century ago by Sampson, nor any single lesion-centered model is sufficient to explain the full biological complexity of the disease [18]. The disease can be seen as a “seed-and-soil” process [14]. The eutopic endometrium appears biologically altered before ectopic implantation, with enhanced proliferative, adhesive, angiogenic, and immune-evasive properties [19,20]. This abnormal endometrial “seed”, characterized by progesterone resistance, aberrant signaling, epigenetic reprogramming, and stem/progenitor cell enrichment, reaches a permissive and actively conditioned peritoneal “soil” in which impaired immune clearance, oxidative stress, and angiogenic signaling facilitate implantation, persistence, and progression [14,18,21].

Endometriotic lesions develop and persist in an inflammatory-redox milieu enriched with activated macrophages, cytokines, growth factors, and oxidative imbalance, all of which support proliferation, angiogenesis, neuroinflammation, and matrix remodeling [22,23,24,25]. In endometriosis, oxidative imbalance is not confined to the lesions themselves, but involves the broader biological environment, including peritoneal fluid, ovarian cortex, and follicular fluid [22,23,25]. Macrophage-driven oxidative stress has been linked to early angiogenesis and lesion establishment, thereby providing a mechanistic connection between immune dysregulation, redox biology, and lesion growth [21].

Alongside analgesia, the main treatment options are surgery and hormonal therapy [26]. Both can be effective, but each has distinct risks and limitations. Surgery may improve pain, lesion burden, and, in selected settings, fertility-related outcomes, yet it is invasive, technically demanding, operator dependent, and not capable of addressing the entire complexity of endometriosis-associated fertility impairment [7,27]. Hormonal agents act through several mechanisms. Depending on the drug class, they inhibit gonadotropin signaling or ovulation, reduce ovarian estrogen production, or exert direct progestogenic effects on ectopic and eutopic tissue, including antiproliferative and pro-apoptotic actions, suppression of local inflammatory and angiogenic signaling, and reduction in intralesional estrogen synthesis [2,28]. These approaches are indispensable in routine care, but they are limited by adverse effects, contraindications, incomplete symptom control, recurrence after discontinuation, and unsuitability for some women seeking conception or long-term tolerability [6,7,28]. The need for therapies that target pathomechanisms beyond endocrine suppression is therefore clinically relevant and increasingly pressing. Potential non-hormonal strategies include anti-inflammatory, anti-angiogenic, antifibrotic, metabolic, ferroptosis-related, immunomodulatory, and dietary interventions [22,28,29].

**Figure 1:**
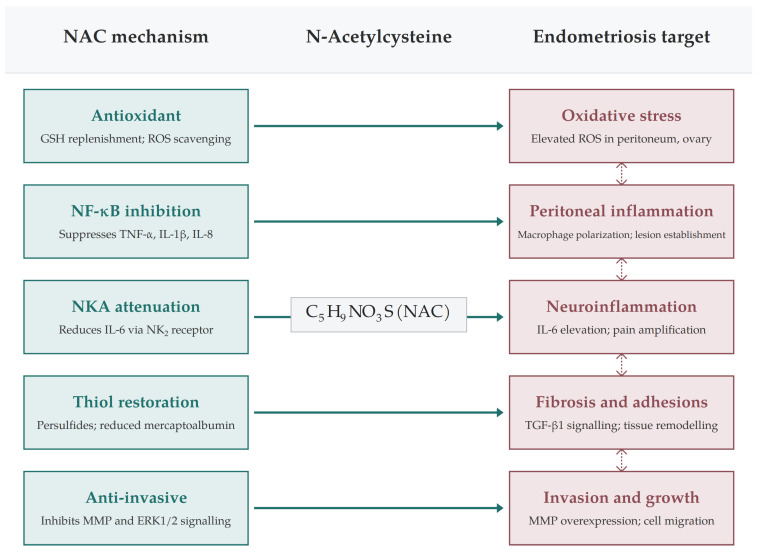
Simplified representation of NAC mechanisms and their potential correspondence with endometriosis pathomechanisms, derived from documented NAC pharmacology. Arrows and connectors indicate proposed dominant relationships; the depicted processes are biologically interconnected and may interact bidirectionally. The strength of supporting evidence in endometriosis varies across domains and is detailed in the Results and Discussion. ERK1/2: extracellular signal-regulated kinase 1/2; GSH: glutathione; IL: interleukin; MMP: matrix metalloproteinase; NAC: N-acetylcysteine; NF-κB: nuclear factor kappa-B; NKA: neurokinin A; NK_2_: neurokinin-2 receptor; ROS: reactive oxygen species; TGF-β1: transforming growth factor beta 1; TNF-α: tumor necrosis factor alpha.

In this context, N-acetylcysteine (NAC) is of particular interest. Beyond its traditionally recognized mucolytic and antidote properties, NAC is a pleiotropic redox-active compound with indirect antioxidant effects through glutathione replenishment, direct thiol-based scavenging capacity, and broader anti-inflammatory, immunomodulatory, matrix-modulating, and potentially antifibrotic actions [30,31]. Its anti-inflammatory activity operates through at least two concentration-dependent pathways: at higher doses, NAC inhibits nuclear factor kappa B (NF-κB) activation and thereby suppresses tumor necrosis factor alpha (TNF-α), interleukin (IL)-1β, and IL-8; at lower doses, it attenuates neurokinin A (NKA) release via a bell-shaped concentration-response relationship, which independently reduces IL-6 production through neurokinin-2 (NK_2_) receptor signaling, a pathway distinct from, and additive to, its antioxidant mechanism [32]. Beyond cytokine regulation, NAC helps restore the systemic thiol pool by reducing cysteinylated extracellular proteins and regenerating reduced mercaptoalbumin, the major antioxidant form of albumin in plasma; in addition, NAC-derived metabolites can generate cytoprotective persulfide and polysulfide species that modulate redox-sensitive signaling [32,33,34]. These pharmacologic properties intersect with several endometriosis-relevant signaling pathways, including oxidative stress, cytokine-driven inflammation, mitogen-activated protein kinase/extracellular signal-regulated kinase (MAPK/ERK) related proliferation, matrix metalloproteinase (MMP) associated invasion, adhesion, pain amplification, and tissue remodeling (Figure 1) [22,28]. These properties, together with its oral availability and long-established safety and tolerability profile, make NAC a plausible drug-repurposing candidate for endometriosis [35,36,37]. The literature on NAC in endometriosis remains limited and heterogeneous, spanning in vitro work, animal models, clinical cohorts, randomized trials, and combination regimens. Accordingly, the primary aim of this systematic review was to evaluate the available evidence on the effects of NAC in endometriosis across clinical and preclinical settings. The secondary aim was to determine which biological pathways and disease dimensions are most consistently implicated, including pain, endometrioma dynamics, recurrence, fertility-related outcomes, inflammation, oxidative stress, migration/invasion, and fibrosis-related processes.

## 2. Materials and Methods

### 2.1. Protocol Registration and Reporting Standard

This systematic review was prospectively preregistered on the Open Science Framework (OSF; https://doi.org/10.17605/OSF.IO/CHKBQ). The review was designed and reported in accordance with the Preferred Reporting Items for Systematic Reviews and Meta-Analyses (PRISMA) 2020 statement [38]. The completed PRISMA 2020 checklist is provided as Supplementary Table S1.

### 2.2. Eligibility Criteria

Human clinical studies, murine and rat in vivo studies, bovine reproductive models, patient-derived ex vivo systems, and mechanistic in vitro studies were included if they investigated NAC, either as an administered intervention or as an experimental probe, in the context of endometriosis, ovarian endometrioma, or experimentally modeled ectopic endometrial disease, and reported at least one endpoint relevant to disease biology or clinical expression. Eligible endpoints included clinical outcomes such as pain, endometrioma size, and postoperative recurrence, as well as fertility-related outcomes and mechanistic endpoints including oxidative stress, inflammatory signaling, migration, invasion, adhesion-related processes, autophagy-related markers, ferroptosis-related mechanisms, and fibrosis-associated pathways. Reviews, editorials, letters without primary data, conference abstracts, and trial protocols were excluded.

### 2.3. Information Sources and Search Strategy

A systematic literature search was conducted in PubMed/MEDLINE, the EBSCOhost platform, including Academic Search Premier, APA PsycArticles, APA PsycInfo, CINAHL, and MEDLINE, and the Bielefeld Academic Search Engine (BASE), from database inception to 24 May 2026.

The PubMed/MEDLINE and EBSCOhost search strategy used the following free-text terms: *“(endometriosis OR endometriotic OR endometrioma OR adenomyosis) AND (n-acetylcysteine OR n-acetyl cysteine OR NAC OR acetylcysteine OR acetyl-cysteine OR N-acetyl-L-cysteine OR N acetyl L cysteine)”*. The BASE search used the all-fields query *“(n-acetylcysteine OR n-acetyl cysteine) AND endometriosis”*. Reference lists of all included reports and relevant reviews were screened for additional records.

No date, language, or species restrictions were applied. Reviews, editorials, and letters without primary data, conference abstracts, and trial protocols were excluded. Preprints reporting primary data were eligible. No dedicated searches of preprint servers, trial registries, or other sources of unpublished data were performed, and study authors were not contacted for additional information.

**Figure 2:**
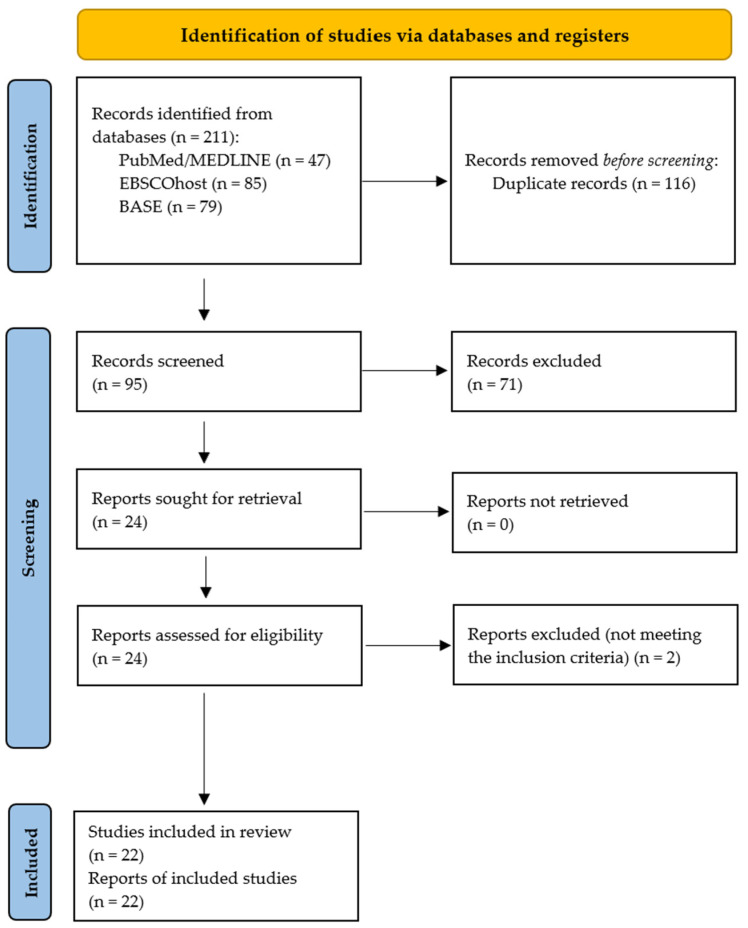
PRISMA 2020 flowchart of study identification and selection.

The PubMed/MEDLINE search retrieved 47 records, the EBSCOhost search retrieved 85 records, and the BASE search retrieved 79 records. After duplicate removal (*n* = 116), 95 records were screened by title and abstract. Seventy-one records were excluded at this stage, and 24 full-text reports were assessed for eligibility. Two full-text reports were excluded because they did not meet the inclusion criteria, leaving 22 studies for the final synthesis. Reference-list screening did not identify additional eligible studies. The selection process is summarized in the PRISMA 2020 flow diagram (Figure 2).

### 2.4. Study Selection

Two reviewers (R.W., S.K.) independently screened titles/abstracts and subsequently assessed full-text reports against the eligibility criteria. Disagreements were resolved by discussion with other authors (A.Y., S.G.V.). No automation tools were used for study selection or data extraction. Twenty-four full-text reports were assessed for eligibility, of which 22 studies met the inclusion criteria. The selection process is summarized in the PRISMA 2020 flow diagram (Figure 2).

### 2.5. Data Extraction

Data were extracted directly from the full texts of the included studies using a structured evidence table. The extracted variables included first author, year, journal, study design, country where applicable, population or experimental model, sample size, diagnostic or modeling approach, NAC regimen (dose, route, duration, and schedule), comparator or control condition, follow-up period where applicable, outcome definitions, and the main quantitative and qualitative findings. For preclinical and mechanistic studies, additional pathway-level variables were captured, including oxidative stress markers, cytokine signaling, matrix remodeling, endoplasmic reticulum stress, autophagy-related readouts, ferroptosis-related mechanisms, and migration or invasion endpoints. Combination regimens containing NAC together with other compounds were recorded separately from NAC-only interventions.

### 2.6. Risk-of-Bias Assessment

Risk of bias was assessed using tools matched to study design. Cochrane Risk of Bias 2 (RoB 2) was used for randomized clinical trials [39]. Risk Of Bias In Non-randomized Studies of Interventions (ROBINS-I) was used for non-randomized clinical intervention studies [40]. Systematic Review Centre for Laboratory Animal Experimentation (SYRCLE) risk-of-bias tool was used for murine and rat in vivo experiments [41]. For mechanistic in vitro and ex vivo components, no universally accepted risk-of-bias instrument is available. We therefore applied a structured appraisal focused on five domains: model relevance and characterization; induction or representation of an endometriosis-related pathological context; NAC intervention reporting, including concentration, exposure time, and translational plausibility; endpoint and pathway specificity; and experimental rigor, including controls, replication, statistical reporting, and masking where reported. For hybrid studies, murine or rat in vivo components were assessed with SYRCLE and cell-based components with this structured appraisal. The appraisal domains and overall criteria are summarized in Supplementary Tables S2 and S3.

Risk-of-bias judgments informed interpretation of the evidence but were not used as exclusion criteria.

### 2.7. Data Synthesis

Given the marked heterogeneity across the included studies with respect to study design, populations, model systems, NAC regimens, comparators, follow-up duration, and outcome definitions, a quantitative meta-analysis was not performed. Instead, the evidence was synthesized narratively. Findings were synthesized across the principal outcome categories that emerged from the literature: pain, endometrioma or lesion burden, postoperative recurrence, fertility-related endpoints, oxidative stress and inflammatory signaling, migration/invasion and matrix remodeling, and fibrosis- or stress-response-related mechanisms. Studies evaluating NAC-containing combination regimens were interpreted separately from NAC-only studies in order to avoid overstating NAC-specific effects.

A formal Grading of Recommendations Assessment, Development and Evaluation (GRADE) assessment was not performed because the review comprised distinct clinical and experimental bodies of evidence that could not be assigned a common certainty rating. Confidence in the findings was addressed through design-specific risk-of-bias assessment and separate interpretation of clinical and nonclinical evidence.

## 3. Results

### 3.1. Study Selection

The PubMed search retrieved 47 records, the EBSCOhost search retrieved 85 records, and the BASE search retrieved 79 records. After duplicate removal (*n* = 116), the remaining 95 records were screened by title and abstract. Twenty-four full-text reports were assessed for eligibility; two were excluded because they did not meet the inclusion criteria, leaving 22 studies for the final synthesis.

### 3.2. Study Characteristics

The main design characteristics of all 22 included studies are summarized in Table 1 [42,43,44,45,46,47,48,49,50,51,52,53,54,55,56,57,58,59,60,61,62,63]. Among them, there were six clinical studies, including two randomized trials and four non-randomized clinical designs [46,52,54,57,58,62]. The other 16 studies formed a heterogeneous preclinical and translational evidence base, including murine and rat in vivo models, rodent nociception assays, patient-derived ex vivo systems, immortalized cell lines, bovine reproductive models, and combined in vivo/in vitro designs [42,43,44,45,47,48,49,50,51,53,55,56,59,60,61,63]. Although the search term “adenomyosis” was included in the search strategy, no study reported NAC data for adenomyosis alone.

The studies were conducted across diverse geographic settings, including Italy, Iran, Turkey, the United States, France, China, Japan, Brazil, Spain, and South Korea. Because of marked heterogeneity in populations, study designs, NAC regimens, co-interventions, follow-up, and outcome definitions, quantitative meta-analysis was not performed and the evidence was synthesized narratively. Since several studies combined animal and in vitro components or linked human biological material with experimental systems, the categories human, animal, and in vitro are not mutually exclusive.

### 3.3. Risk of Bias Across Studies

Among the two randomized clinical trials, both were judged as raising some concerns, although for different reasons. In the study by Asgari et al. [57], randomization and assessor blinding were described, but no placebo was used, participants were aware of treatment allocation, and one of the main outcomes was pain, which remained vulnerable to performance and detection bias. In the study by Heshmati et al. [62], the placebo-controlled design was methodologically stronger, but the sample was small, three participants in the NAC arm were excluded after randomization, and most downstream molecular endpoints remained statistically non-significant. Among non-randomized clinical studies, the risk of bias was higher. The study by Porpora et al. [46] was based on treatment acceptance versus refusal and was therefore prone to selection bias and confounding. Anastasi et al. [58] and Lete et al. [52] conducted uncontrolled prospective studies, while Fadin et al. [54] used a historical-control design, making these studies particularly vulnerable to regression to the mean, co-intervention, and expectation effects. Among the animal and hybrid in vivo studies, most judgments were some concerns or unclear because reporting of sequence generation, allocation concealment, random housing, and blinding was limited, although several endpoints were objective and incomplete outcome data were generally not a major concern. Across mechanistic in vitro studies, all studies raised some concerns, chiefly because masking was not reported and translational relevance was constrained by model-dependent systems and, in some studies, supraphysiological or otherwise difficult-to-translate NAC concentrations. The mechanistic findings recurred across several models but remained model-dependent, whereas the clinical evidence was methodologically limited. Detailed design-specific judgments are summarized in Table A1.

### 3.4. Effects

#### 3.4.1. Pain Outcomes

Pain outcomes are summarized in Table 2. Six studies reported clinical or translational pain-related outcomes. Four reported improvement, but the positive clinical evidence came from one non-randomized comparative cohort and one uncontrolled single-cohort study [46,58] or from combination regimens in which the contribution of NAC alone could not be isolated [52,54]. Porpora et al. and Anastasi et al. used the same intermittent oral regimen and both observed clinically relevant improvement in distinct pain domains, with Anastasi et al. additionally documenting reduced NSAID use [46,58]. The only randomized postoperative study, by contrast, did not demonstrate an additive analgesic effect over oral contraceptive therapy alone [57]. Ray et al. should be interpreted separately as mechanistic evidence for oxidation-sensitive nociception rather than as direct clinical pain evidence [49].

#### 3.4.2. Lesion Burden, Lesion Activity, and Endometrioma Size Outcomes

Lesion burden, lesion activity, and endometrioma size outcomes are summarized in Table 3. In NAC treatment models, murine and rat data showed reductions in lesion mass, implant area, or lesion activity, while the Erastin ferroptosis model showed a different pattern in which NAC-mediated ROS scavenging attenuated Erastin-induced lesion regression [44,45,47,61]. In the study by Pittaluga et al., this corresponded to an average endometrioma weight of 29 ± 4 mg in NAC-treated samples versus 74 ± 9 mg in controls (*p* < 0.01) [45]. In the human observational cohort studied by Porpora et al., mean cyst diameter decreased in the NAC group (−1.53 ± 11.43 mm) and increased in untreated controls (6.62 ±16.14 mm) over 3 months; volume evolution was likewise more favorable in the NAC group (with a between-group difference of 28.9 mL), and 24 women cancelled laparoscopy because of cyst decrease/disappearance, pain reduction, and/or pregnancy [46]. Anastasi et al. also reported a modest but statistically significant reduction in mean endometrioma size in an uncontrolled prospective cohort [58]. By contrast, Asgari et al. found no significant reduction in post-surgical recurrence rate under adjunct NAC plus oral contraceptive therapy [57]. In the study by Onalan et al. [47], the NAC result reflects a within-group pre/post reduction in implant area rather than a direct NAC-versus-control endpoint comparison.

#### 3.4.3. Fertility Outcomes

Fertility outcomes are summarized in Table 4. The fertility findings remain preliminary. The pregnancy data reported by Porpora et al. [46] and Anastasi et al. [58] were directionally favorable, but neither study was designed or controlled as a fertility trial. In the study by Anastasi et al., the fertility findings included 39 spontaneous pregnancies and 6 pregnancies achieved through ART among 52 women with reproductive desire [58]. The placebo-controlled trial by Heshmati et al. found trends favoring NAC for oocyte and embryo parameters, but the differences were not statistically significant [62]. The two translational studies by Giorgi et al. demonstrated that NAC improved oocyte and embryo outcomes in bovine systems exposed to follicular fluid from women with mild endometriosis, providing mechanistic support for a protective effect against oxidative injury [51,56].

#### 3.4.4. Inflammatory, Oxidative Stress, and Molecular Markers

Inflammatory, oxidative stress, and molecular marker outcomes are summarized in Table 5. In primary endometriotic cells, NAC reduced H_2_O_2_ production and proliferation and normalized the pERK/ERK ratio [44]. In mice, NAC reduced COX-2 gene and protein expression, decreased MMP-9 activity, reduced Ki-67 labeling, and increased the localization of E-cadherin at cell–cell borders [45]. In two rat studies, NAC was associated with lower inflammatory cytokine levels under different experimental conditions. Serum TNF-α decreased significantly within the NAC-treated group in Onalan et al., whereas the decrease in peritoneal TNF-α was not significant [47]. In the Erastin model, NAC co-treatment reduced serum TNF-α, IL-6, and IL-1β relative to Erastin alone (*p* < 0.001) [61]. In a separate study using female Wistar rats, NAC reduced ROS levels and downregulated LC3 and Beclin-1 [53]. In human endometriotic endothelial cells, the combination of NAC, alpha-lipoic acid, and bromelain prevented TNF-α-induced VCAM1 upregulation, whereas the individual components were not significant [50]. In an inflamed endometrial epithelial model, a formulation containing NAC, quercetin, and turmeric reduced IL-6 with increasing concentration; TNF-α decreased at lower concentrations but showed a partial rebound at 380 µg/mL [54]. Because both studies evaluated multi-component formulations, these effects cannot be attributed to NAC alone. In 12Z cells, NAC suppressed iron-induced migration and MMP-2/-9 expression; NF-κB involvement was examined separately with Bay-11-7082, and direct inhibition of NF-κB by NAC was not demonstrated [55]. In another 12Z cell study, NAC reduced β-tubulin labeling and increased ER-tracker and GRP78 labeling, whereas these changes were not significant in HESC cells [59]. A subsequent study found that NAC reduced 12Z cell proliferation and increased ER-tracker and phosphorylated inositol-requiring enzyme 1 (p-IRE1) labeling. IFN-γ co-treatment further enhanced the antiproliferative effect, whereas p-IRE1 labeling was highest with IFN-γ alone [60]. In Erastin-treated hEM15A cells, NAC reduced ROS, SLC1A5 mRNA, c-Myc expression, Glu, Gln, Fe^2+^, and MDA, while increasing GSH and GPX4 [61]. In an uncontrolled single-cohort study, mean serum CA125 decreased from 45.55 ± 26.5 to 35.6 ± 24.2 U/mL (*p* = 0.001) [58]. In the randomized trial, serum TAC increased significantly after NAC, whereas the increase in SOD was not significant; Bcl-2 expression was higher and Bax and Caspase-3 expression lower than with placebo, but none of these gene-expression differences reached statistical significance [62]. Under H_2_O_2_ challenge in 12Z and Ishikawa 2D/3D cultures, NAC restored viability and reduced lipid and protein oxidation but did not prevent DNA damage [63].

#### 3.4.5. Cellular Proliferation, Migration, and Invasion

Cellular proliferation, viability, migration, invasion, and stress-response outcomes are summarized in Table 6. NAC demonstrated antiproliferative effects on endometrial and endometriotic stromal cells across the in vitro studies, with dose-dependent inhibition ranging from 35% to 95% in the assays that quantified this outcome [42,43]. However, the concentrations required (5–30 mM) substantially exceed physiological plasma concentrations achievable with oral dosing, limiting direct translational inference. Matsuzaki and Darcha found that NAC reduced proliferation but did not significantly alter migration, invasion, or collagen-gel contraction. Among the fibrosis-related genes examined, only alpha-smooth muscle actin (αSMA) mRNA decreased, and only in endometrial stromal cells, whereas epigallocatechin-3-gallate (EGCG) showed broader inhibitory effects across these endpoints in parallel experiments [48]. The available evidence most consistently supports antiproliferative and redox-stress-modulating effects, while anti-invasive, antifibrotic, ferroptosis-related, and oxidative-damage endpoints remain more model-dependent. In patient-derived endometriotic cells, NAC normalized a significantly elevated pERK/ERK ratio alongside dose-dependent reductions in H_2_O_2_ and proliferation, implicating ROS-driven ERK1/2 activation as a specific pathway in primary endometriotic cells [44]. In 3D spheroid culture under oxidative challenge, 12Z endometriotic cells showed 72% glutathione depletion—a vulnerability not apparent in 2D models—and NAC restored viability and reduced lipid and protein oxidation but did not prevent DNA strand damage [63], indicating that cytoprotective and genomic endpoints are dissociable.

#### 3.4.6. Adverse Events and Tolerability

Among the human studies, NAC monotherapy was generally well tolerated. Porpora et al. reported no adverse reactions at intermittent dosing of 1800 mg/day for 3 consecutive days per week [46], and Anastasi et al. observed no side effects [58]. In the randomized trial by Heshmati et al., 2 participants discontinued treatment because of gastrointestinal complaints and 3 participants in total were excluded after randomization from the NAC arm [62]. In the LEAP study, 52 women withdrew overall, including 27 because of pregnancy, 12 because of uncontrolled pain, and 13 because of side effects, mainly nausea and vomiting [52]. Fadin et al. reported good tolerability of the lower-dose combination formulation [54].

### 3.5. Evidence Summary

Of the 22 included studies, most reported at least one NAC-associated biological or clinical effect, but these findings cannot be pooled because the studies addressed different questions. The clinical evidence was restricted to six studies, including two randomized trials and four non-randomized or uncontrolled designs, with follow-up ranging from two to six months and NAC doses from 150 mg/day in combination regimens to 1800 mg/day in intermittent monotherapy. Preclinical evidence comprised seven animal or hybrid in vivo studies and nine mechanistic or translational in vitro studies conducted across murine, rat, bovine, and human cell-based systems. Interpretation across outcome categories was limited by design-related constraints, particularly the absence of concurrent controls in most clinical studies, the restricted endpoint scope of the postoperative randomized trial, and the use of difficult-to-translate NAC concentrations in several cell-based models.

## 4. Discussion

Endometriosis is a chronic inflammatory condition sustained by estrogen-dependent lesion growth, oxidative imbalance, macrophage activation, angiogenesis, immune dysregulation, and tissue remodeling [18,22,45]. Oxidative stress is not confined to ectopic lesions but extends to the peritoneal environment, ovarian cortex, and follicular fluid [23]. NAC was approved by the Food and Drug Administration in 1963; its anti-inflammatory and antioxidative properties are utilized in chronic inflammatory and fibrotizing respiratory diseases, including chronic obstructive pulmonary disease, bronchial asthma, idiopathic lung fibrosis, and lung silicosis [30]. These combined anti-inflammatory and redox-modulating properties make NAC a biologically plausible candidate for non-hormonal supportive intervention in endometriosis. In addition, more than 60 years of experience with NAC as a mucolytic drug and paracetamol antidote support its favorable safety profile [35,36,37]. Across the broader NAC literature, adverse effects are uncommon and largely route-dependent; gastrointestinal symptoms (heartburn, nausea, flatulence) represent the most frequently reported events with oral administration, while serious adverse effects are rare and primarily associated with intravenous administration at very high doses [30,35].

The first experience with NAC in endometriosis came from cell-based models in the early 2000s. Foyouzi et al. showed that NAC inhibited proliferation of endometrial stromal cells in a dose-dependent manner, with suppression reaching 52–85% at 10–30 mM (thymidine incorporation) and 95% inhibition at 30 mM (MTT assay) [42]. Wu and Guo confirmed a similar redox-sensitive antiproliferative effect, with 35–50% inhibition at 10–30 mM and 63% suppression of H_2_O_2_-induced proliferation in an immortalized stromal cell line [43]. However, these concentrations substantially exceed plasma levels achievable with oral dosing, a limitation that constrains direct extrapolation to clinical settings. This “concentration gap” between in vitro and in vivo studies is common in pharmacology. For instance, in airway epithelial cell models, NAC concentrations of 16 µM and 35 µM have been used to approximate plasma levels reported after oral 600 mg and 1200 mg dosing, whereas 1.6 mM and 5 mM represent commonly applied in vitro concentrations; low micromolar concentrations required prolonged exposure for sustained antioxidant and anti-inflammatory effects, while higher concentrations produced stronger acute effects [64]. In a paraquat-induced oxidative stress model, approximately 10 mM extracellular NAC was required for about 50% suppression of intracellular ROS, a concentration that could not be translated into a clinically feasible intravenous regimen [65]. Oral NAC has low bioavailability, generally below 10%, and systematic pharmacokinetic, pharmacodynamic, and dose-ranging data remain limited [30,35,36]. High-concentration in vitro studies can identify redox-sensitive pathways, antiproliferative effects, or stress-response mechanisms, but cannot establish equivalent exposure in human endometriotic lesions. At the same time, their biological meaning depends on the experimental system. Dose-dependent effects at 6.4–10 mM were observed in primary endometriotic cells; in the same study, NAC also reduced lesion pathology in a human-tissue xenograft model treated with 60 mg per mouse [44]. In 3D spheroid culture, oxidative challenge depleted glutathione in 12Z cells by 72%, a vulnerability not apparent in parallel 2D cultures, indicating that culture geometry can change the apparent NAC effect window [63].

Despite heterogeneous study designs, the molecular data converge on several further pathways through which NAC may act: reduction in ROS and oxidative stress [53,62], attenuation of inflammatory mediators including TNF-α and COX-2 [45,47], suppression of iron-induced migration and MMP-2/-9 expression [55], and modulation of ER-stress-related pathways in endometriotic cell models [59,60]. By contrast, Matsuzaki and Darcha found an antiproliferative effect of NAC but no significant effect on migration, invasion, or collagen–gel contraction and only a limited effect on fibrosis-related gene expression [48].

Across the studies included in this review, the label “oxidative stress” covers measurements that do not all describe the same biological process. Reactive oxygen species (ROS) probe signals, reduced glutathione/glutathione disulfide (GSH/GSSG) ratios, total glutathione content, lipid peroxidation products, protein carbonylation, and deoxyribonucleic acid (DNA) oxidation or damage capture different aspects of redox state and biomolecular injury. Cytokine release, matrix metalloproteinase (MMP) activity, endoplasmic reticulum (ER) stress, autophagy, apoptosis, migration, and invasion are downstream or intersecting processes whose redox dependence must be demonstrated experimentally. These differences help explain the inconsistent biomarker literature in endometriosis. Total antioxidant capacity (TAC) summarizes aggregate reducing capacity in a given matrix, while superoxide dismutase (SOD) and glutathione peroxidase (GPx) activity reflect antioxidant defense and enzymatic adaptation. A small diagnostic study proposed combined SOD/GPx testing as a possible preoperative biomarker [66], whereas erythrocyte SOD, erythrocyte GPx, serum hexanoyl lysine (HEL), and peritoneal-fluid HEL did not distinguish infertile patients with endometriosis from infertile controls in a prospective cohort [67]. TAC, SOD, GPx, and phenylalanine also differed between mild and severe endometriosis in one study, while interleukin-6 (IL-6) did not [68]. Systemic antioxidant markers therefore provide only partial information on redox adaptation and cannot serve as interchangeable surrogates of lesion-level oxidative injury. Thiobarbituric acid reactive substances (TBARS) and malondialdehyde (MDA) are commonly used as lipid-peroxidation readouts, although their specificity is lower than that of chromatographic or mass-spectrometric methods [69].

Preclinical studies support several related mechanisms that require further clinical testing. In a murine model NAC reduced endometrioma mass by approximately 60%, with concurrent decreases in Ki-67 labeling by 54%, COX-2 expression, and MMP-9 activity by more than 60%, and with a shift in E-cadherin localization from cytoplasm to cell–cell junctions indicative of a more differentiated phenotype [45]. In a randomized rat autotransplantation model, NAC caused significant reductions in implant area and serum TNF-α, with a trend toward lower peritoneal TNF-α levels [47]. Both in vivo studies suggested that NAC is capable of reducing lesion burden by targeting proliferation, inflammation, and matrix remodeling. The xenograft component in the study by Ngô et al., in which nude mice bearing implants of human ovarian endometrioma tissue were treated with NAC, extended these findings to an in vivo model using human endometriotic tissue. NAC reduced the pathology score from 2.0 ± 0.25 in phosphate-buffered saline (PBS)-treated controls to 1.19 ± 0.13 (*p* < 0.025), with NAC-treated lesions showing fibrotic and avascular histology [44].

In the hemorrhagic, iron-rich peritoneal microenvironment of endometriosis, Woo et al. showed that iron overload stimulated ROS production, migration, and MMP-2/-9 expression in 12Z endometriotic cells and that NAC suppressed iron-induced migration and MMP-2/-9 expression [55]. Lu et al. extended the mechanistic observation to ROS-associated autophagy-related signaling: in a rat endometriosis model, NAC reduced ROS levels and downregulated the autophagy markers LC3 and Beclin-1, suggesting that its action extends to cellular stress-adaptation responses beyond direct antioxidant activity [53]. The Karakoç studies further showed that NAC, at its determined IC50, reduced proliferation and migration in the 12Z endometriotic cell line while inducing endoplasmic reticulum stress-related changes including decreased β-tubulin and increased GRP78 expression; in a cytokine-rich environment with IFN-γ co-treatment, the antiproliferative effect was enhanced, supporting a context-dependent pattern of NAC activity [59,60]. Notably, these effects occurred at an IC50 several orders of magnitude below the millimolar concentrations required in other NAC cell models, which—if independently verified—would place these results closer to clinically achievable exposure levels. Matsuzaki and Darcha found an antiproliferative effect of NAC but no significant effect on migration, invasion, or collagen-gel contraction and only a limited effect on fibrosis-related gene expression. In this model, NAC therefore showed a clearer antiproliferative than antifibrotic or anti-invasive effect. In the same study, EGCG showed broader activity across these endpoints and was the only compound tested in the xenograft model [48]. The contrast with migration-related effects observed in other cell models indicates marked model dependence [55,59,60]; whether this has clinical relevance for deep or fibrotic disease remains unknown.

Ma et al. demonstrated a further dimension of mechanistic complexity. In hEM15A cells, Erastin increased ROS, SLC1A5, and c-Myc expression, altered glutamine metabolism, and reduced GSH, while NAC reversed several of these ferroptosis-related changes. In the rat endometriosis model, Erastin reduced lesion volume from 94.8 ± 13.5 to 21.2 ± 2.0 mm^3^ and the pelvic adhesion score from 8.3 ± 0.6 to 2.7 ± 0.2 (*p* < 0.01). Co-administration of NAC at 250 mg/kg partially attenuated these effects, increasing lesion volume to 34.9 ± 2.6 mm^3^ and the adhesion score to 4.3 ± 0.4 (*p* < 0.05 versus Erastin alone). This mechanistic duality—antioxidant cytoprotection versus antagonism of ferroptosis-directed lesion suppression—represents a potential constraint on the use of NAC in contexts where ROS-dependent cell death is the intended mechanism of action [61].

Pain is the central clinical manifestation of endometriosis. Ray et al. provided mechanistic evidence that oxidatively modified lipoproteins from the peritoneal fluid of women with endometriosis induced nociceptive responses in rodent models, and that NAC suppressed the pain-inducing activity of these oxidized mediators [49]. Pain in endometriosis does not map linearly onto lesion size but arises from inflammatory signaling, oxidative injury, prostaglandin biology, peripheral neuroangiogenesis, and sensitization [70,71]. A therapy targeting redox-sensitive pain biology may therefore improve symptoms independently of structural disease modification. This is consistent with findings from a recent meta-analysis of antioxidant supplementation in endometriosis, which found benefits for several specific pain domains and oxidative stress markers (but no clear effect on clinical pregnancy rate), despite heterogeneity across antioxidant classes and trial designs [72].

Fertility-related findings currently support biological plausibility, but are not yet sufficient to support clinical translation. The two studies by Giorgi et al. suggest that NAC can partly mitigate the adverse reproductive effects of an endometriosis-associated follicular milieu, but the pattern of benefit argues against a general fertility-enhancing effect [51,56]. This selective responsiveness is biologically credible in the context of oxidative stress and is further supported by experimental work linking NAC to redox-sensitive oocyte protection [73]. In contrast to these indirect models, the only human randomized trial did not demonstrate significant improvement in oocyte or embryo outcomes despite evidence of systemic antioxidant target engagement [62]. The bench-to-bedside gap therefore remains real. The broader literature on antioxidant supplementation in female subfertility is similarly uncertain and heterogeneous [74].

The six clinical studies did not investigate the same therapeutic question. Porpora et al. and Anastasi et al. evaluated NAC as a direct intervention in active, non-postoperative disease [46,58]. Asgari et al. tested it as an add-on to continuous oral contraceptives after laparoscopic surgery [57]. Lete et al. [52] and Fadin et al. [54] used NAC within fixed multi-ingredient combinations, and Heshmati et al. conducted a placebo-controlled trial focused on molecular endpoints in infertile women [62]. Interpreting them as a single body of evidence would obscure the clinical differences between settings.

The placebo-controlled trial by Heshmati et al. provided preliminary direct human evidence of an in vivo antioxidant effect of NAC. In 11 NAC-treated and 14 placebo-analyzed infertile women with endometriosis receiving 1200 mg/day for six weeks, TAC increased significantly from baseline in the NAC group, whereas the increase in SOD was not significant [62]. In the NAC group, granulosa cell expression of Bcl-2, Bax, and Caspase-3 showed favorable non-significant trends, consistent with a positive trend for embryo quality. The within-group TAC increase is compatible with a systemic antioxidant effect, but the study was too small to establish clinically relevant differences in reproductive endpoints.

Lete et al. enrolled 398 women with endometriosis-associated pelvic pain in a multicenter open-label study using a fixed combination of NAC 600 mg, alpha-lipoic acid 200 mg, bromelain 25 mg, and zinc 10 mg, administered as two tablets daily for six months; the study reported a continuous decline in mean VAS pain scores and NSAID use [52]. Fadin et al. used a lower-dose NAC combination (150 mg with quercetin and turmeric) for two months in a small historically controlled study and reported substantial reductions in all three pain domains [54]. Agostinis et al. demonstrated anti-inflammatory and anti-lesion effects of a NAC/alpha-lipoic acid/bromelain combination in SCID mice and in endometriotic endothelial cell models [50]. The findings encourage further studies on multi-target approaches including NAC. However, the concurrent administration of biologically active compounds such as alpha-lipoic acid, bromelain, or curcumin precludes the attribution of these effects solely to NAC. Consequently, these studies provide evidence for the therapeutic potential of antioxidant combinations, but do not allow us to define the individual contribution of NAC to the observed effects.

Porpora et al. enrolled 92 women (47 cases and 45 controls) with ultrasound-confirmed ovarian endometrioma scheduled for laparoscopy and allocated them according to treatment acceptance to NAC (600 mg three times daily on three consecutive days per week) or no treatment for three months [46]. Over this period, mean cyst diameter and volume decreased in the NAC group (while increasing in controls) and pain improved significantly in all assessed domains. Most strikingly, twenty-four NAC-treated women cancelled scheduled laparoscopy due to cyst regression, pain reduction, or pregnancy, compared with one in the control group. Anastasi et al. applied the same regimen prospectively to 120 women with endometriosis and documented highly significant improvements in dysmenorrhea, dyspareunia, and chronic pelvic pain, together with reductions in NSAID use, mean endometrioma diameter, and CA125 [58]. Both studies lacked randomization, and Anastasi et al. had no control group. Nonetheless, the NAC regimen was clearly specified, and the observations of both studies were consistent regarding clinically relevant and measurable endpoints.

The randomized trial by Asgari et al. tested whether adding NAC (600 mg three times daily for three consecutive days per week, for three months) to continuous low-dose oral contraceptives (COC; 30 µg ethinyl estradiol + 0.15 mg levonorgestrel) after conservative laparoscopic surgery for “stage IV” (no staging system indicated) ovarian endometrioma improved recurrence rate or pelvic pain compared with COC alone [57]. Neither recurrence at six months nor pain reduction showed a significant between-group difference. Of note, this negative add-on trial in a postoperative, hormonally suppressed cohort did not include an NAC-only arm. Several additional limitations further complicate its interpretability. The study is described as double-blind, but no placebo was available and participants were aware of their treatment allocation; blinding was restricted to outcome assessment and data analysis [57]. Pain was recorded as a single undifferentiated global pelvic VAS without separation of dysmenorrhea, dyspareunia, and chronic non-cyclic pain. The endpoint captured only sonographic recurrence of ovarian endometrioma larger than 20 mm, not considering superficial peritoneal or deep infiltrating disease. Operative radicality is not documented, and the disease staging classification is not specified; if a revised American Society for Reproductive Medicine (rASRM) classification was used, this would not adequately depict deep disease burden [22]. This study illustrates that randomization is not a self-sufficient guarantee of study quality. Even randomized studies may yield weak or misleading inferences when implementation, endpoint architecture, or reporting are flawed [75,76]. In the present context, interpretation of the three clinical studies evaluating the therapeutic benefit of NAC is difficult, because consistent observational evidence contrasts with flawed, although randomized, evidence. A Cochrane methodology review found no difference or only a very small average difference between effect estimates from randomized and observational studies, but stressed that population, intervention, comparator, outcome definitions, heterogeneity, and risk of bias must be considered when study designs yield different results [77]. In the present review, the postoperative randomized trial evaluated NAC as an add-on to continuous oral contraceptive therapy after conservative surgery, whereas the observational monotherapy studies concerned active, non-postoperative disease and endometrioma behavior. These bodies of evidence should therefore be interpreted separately.

This systematic review is the first evidence synthesis of NAC in endometriosis. It integrates mechanistic and clinical perspectives. Given the disease burden and chronicity, diagnostic delay and narrow scope of non-surgical therapeutic options, this systematic synthesis on a biologically plausible and potentially useful agent fills one of dozens of gaps in the “undone science” and “willful ignorance” of endometriosis [78].

### Limitations

Only six clinical studies were eligible, and only two were randomized. Follow-up was short, outcome definitions differed, and the studies addressed distinct clinical settings, including active symptomatic disease, ovarian endometrioma, postoperative suppression, infertility treatment, and multi-component interventions. These differences precluded quantitative synthesis. In combination studies, the contribution of NAC could not be separated from that of the co-administered compounds. Several mechanistic studies also used supraphysiological concentrations or model-specific endpoints that limit extrapolation to clinical dosing.

The review did not apply a formal certainty-of-evidence method across the complete evidence base. A single overall rating would have combined randomized trials, observational cohorts, murine and rat experiments, patient-derived ex vivo systems, and cell-based studies that addressed different questions. Confidence was therefore assessed through design-specific risk-of-bias methods and separate interpretation of clinical and nonclinical findings, although no overall certainty rating was assigned.

The search covered PubMed/MEDLINE, five databases available through EBSCOhost, BASE, and the reference lists of included reports and relevant reviews. Embase, Scopus, and Web of Science were not searched, and the possibility of missed records cannot be excluded.

Future trials should use adequate sample sizes, placebo control, NAC monotherapy, active non-postoperative disease cohorts, separate assessment of pain domains, standardized endometrioma measurements, and pharmacodynamic oxidative stress markers in clinically relevant compartments. The most consistently positive non-postoperative findings were observed with oral NAC 600 mg three times daily on three consecutive days per week for three months in women with active symptomatic disease or ovarian endometrioma [46,58]. Tolerability was good, with mild gastrointestinal symptoms as the main adverse effect [30,35]. This evidence remains non-randomized; any clinical use should remain off-label, individualized, and accompanied by structured follow-up of pain and endometrioma size.

## 5. Conclusions

NAC has shown a plausible preclinical profile across murine, rat, bovine, patient-derived, and cell-based systems, with effects on redox-sensitive proliferation, inflammatory signaling, lesion activity, selected oxidative-injury endpoints, and context-dependent migration-related behavior. This activity appears strongest in proliferative, inflammatory, and redox-sensitive domains, whereas antifibrotic, anti-invasive, ferroptosis-related, and oxidative-genomic effects are more model-dependent. Notably, NAC’s antioxidant activity antagonizes ROS-dependent ferroptotic lesion suppression, and its cytoprotective effects do not extend to DNA strand integrity under sustained oxidative challenge in 3D endometriotic cell culture. The clinical literature suggests potential benefit for pain and endometrioma dynamics in active non-postoperative settings under intermittent oral monotherapy, but this is based on non-randomized evidence. In contrast, the only randomized trial, which tested a postoperative add-on design against an oral contraceptive formulation, was negative; however, it presented important limitations in comparator choice, surgical reporting, and outcome construction. Whether NAC can be repurposed for active non-postoperative endometriosis, and whether its mechanistic effects on oxidative stress and proliferation translate into clinical benefit, remain to be established in adequately designed, placebo-controlled, phenotype-stratified randomized trials.

## Figures and Tables

**Table 1:** Characteristics of all 22 included studies.

Study	Study Type	Model System	NAC Dose/Route	Duration	Co-Treatments	Primary Endpoints
Foyouzi et al., 2004 [42]	Experimental (in vitro)	Endometrial stromal cells from women with and without endometriosis	0.3–30 mM in culture	48 h (thymidine)/96 h (MTT)	None	Cell proliferation (thymidine incorporation, MTT)
Wu and Guo, 2006 [43]	Experimental (in vitro)	YHES immortalized stromal cell line	10 and 30 mM in culture	48 h, with long-term follow-up in selected assays	H_2_O_2_ in selected experiments	Proliferation, PR-A/PR-B, AR, Fas, FasL expression
Ngô et al., 2009 [44]	Ex vivo/in vitro + murine xenograft	Patient-derived cells; nude mice	6.4/10 mM in vitro; 60 mg/mouse i.p.	18–48 h; 4 weeks in vivo	Danazol and RU-486 comparator arms	ROS, H_2_O_2_, GSH, SOD/catalase, proliferation, pERK/ERK, lesion score
Pittaluga et al., 2010 [45]	Experimental (murine)	BALB/c mouse model (*n* = 60)	44 mg/kg/day by oral gavage	5 days/week for 3 weeks	None	Endometrioma weight (mg), Ki-67, E-cadherin, COX-2, MMP-9
Porpora et al., 2013 [46]	Observational cohort	Human clinical study (92 women with ovarian endometrioma)	600 mg orally three times daily, 3 days/week	3 months	None	Cyst diameter and volume, pain, pregnancy, surgery cancellation
Onalan et al., 2014 [47]	Prospective randomized rat study	Rat autotransplantation model (*n* = 40)	200 mg/day orally	3 weeks treatment after lesion establishment	Comparator arms: amifostine, leuprolide, untreated control	Implant area, serum TNF-α, peritoneal TNF-α
Matsuzaki and Darcha, 2014 [48]	Experimental (in vitro) *	Patient-derived endometrial/endometriotic stromal cells	5 or 10 mM in culture	24–48 h, assay-specific	EGCG comparator; ±TGF-β1	Proliferation, migration, invasion, fibrosis markers
Ray et al., 2015 [49]	Hybrid mechanistic/translational study	Human PF/LDL model; mouse thermoregulation; rat Hargreaves assay; Ishikawa cells	1 mM during LDL oxidation; no systemic NAC dosing	Mechanistic exposure model; pain assays up to 360 min	Vitamin E, indomethacin in selected experiments	Oxidized lipoproteins, eicosanoids, rat nociception, mouse thermoregulation, gene expression
Agostinis et al., 2015 [50]	Experimental (in vivo + in vitro)	SCID mice plus human endometriotic endothelial cell models	1000 µg/mL in vitro; 250 mg/kg/day orally in vivo	21 days in vivo	Alpha-lipoic acid, bromelain	Cyst number and size, VCAM1, apoptosis
Giorgi et al., 2016 [51]	Experimental (in vitro)	Bovine oocytes exposed to human follicular fluid from women with mild endometriosis or controls	1.5 mM in IVM medium	22–24 h culture	L-carnitine in some groups	Meiotic normality of MII oocytes
Lete et al., 2018 [52]	Multicenter open-label non-comparative clinical trial	Human clinical study (*n* = 398)	NAC 600 mg + alpha-lipoic acid 200 mg + bromelain 25 mg + zinc 10 mg, 2 tablets/day orally	6 months	Alpha-lipoic acid, bromelain, zinc (fixed combination)	Pelvic pain (VAS), rescue analgesic use
Lu et al., 2020 [53]	Experimental (rat)	Female Wistar rats (10/group)	200 mg/kg by peritoneal perfusion	7 days treatment plus 21 days convalescence	Catalase comparator; normal saline comparator	ROS, LC3, Beclin-1, lesion histology
Fadin et al., 2020 [54]	Open-label single-center study versus historical control	Human clinical study (*n* = 33)	150 mg/day orally	2 months	Quercetin 200 mg + turmeric 210 mg (fixed combination)	Pain (NRS), NSAID use, inflammatory cytokines
Woo et al., 2020 [55]	Experimental (in vitro + tissue analysis)	12Z human endometriotic cells with supporting human and murine tissue analyses	5 mM NAC in vitro	24 h in vitro migration experiments	None	Migration, MMP-2/-9, ROS, NF-κB, EMT-related changes
Giorgi et al., 2021 [56]	Experimental (in vitro)	Bovine embryo model exposed to human follicular fluid from women with mild endometriosis or controls	1.5 mM in culture medium	22–24 h maturation plus embryo culture	L-carnitine in some groups	Embryo cleavage, blastocyst formation, hatching rate
Asgari et al., 2022 [57]	Randomized postoperative clinical trial	Human clinical study (*n* = 100 women with stage IV endometriosis after conservative laparoscopy)	600 mg orally three times daily, 3 days/week	3 months NAC treatment; 6 months follow-up under COC	Low-dose oral contraceptive	Endometrioma recurrence, pelvic pain (VAS)
Anastasi et al., 2023 [58]	Prospective single-cohort study	Human clinical study (*n* = 120)	600 mg orally, 3 tablets/day, 3 consecutive days/week	3 months treatment; 6 months fertility follow-up	None	Pain (VAS), NSAID use, endometrioma size, CA125, pregnancy
Karakoç, 2023 [59]	Experimental (in vitro)	12Z and HESC cell lines	IC50-based NAC concentration (3.87 × 10^−9^ M)	96 h impedance monitoring; wound-healing measurements at 0, 24, and 48 h	None	Proliferation, migration, β-tubulin, ER stress
Karakoç et al., 2025 [60]	Experimental (in vitro)	12Z and HESC cell lines with cytokine-context modulation	IC50-based NAC concentration (3.87 × 10^−9^ M) in culture medium	96 h monitoring	IFN-γ, TNF-α, IL-6	Proliferation, apoptosis-related assays, ER stress, metabolomics
Ma et al., 2025 [61]	In vitro + rat model	hEM15A cells; rat endometriosis model	5 mM in vitro; 250 mg/kg i.p.	24 h; 28 days	Erastin; SLC1A5 modulation	ROS/c-Myc/SLC1A5, Gln/Glu, GSH, Fe^2+^, MDA, GPX4, lesion volume
Heshmati et al., 2026 [62]	Double-blind placebo-controlled randomized clinical trial	Human clinical study (*n* = 28 randomized; 11 NAC and 14 placebo analyzed)	1200 mg/day orally	6-week intervention	Placebo comparator	TAC, SOD, Bcl-2/Bax/Caspase-3, oocyte/embryo quality, pregnancy
Coelho et al., 2026 [63]	In vitro	12Z/Ishikawa 2D/3D cultures	5 mM in 2D; 1.25 mM in 3D	24 h/assay-specific	H_2_O_2_ challenge	Viability, ROS, GSH/GSSG, lipid/protein oxidation, DNA damage

* NAC was evaluated in vitro only; the xenograft experiments tested EGCG. Abbreviations: 2D, two-dimensional; 3D, three-dimensional; AR, androgen receptor; ART, assisted reproductive technology; CA125, cancer antigen 125; COC, combined oral contraceptives; EGCG, epigallocatechin-3-gallate; EMT, epithelial–mesenchymal transition; ER, endoplasmic reticulum; FasL, Fas ligand; Fe^2+^, ferrous iron; Gln, glutamine; Glu, glutamate; GPX4, glutathione peroxidase 4; GSH, glutathione; GSH/GSSG, reduced glutathione/glutathione disulfide ratio; H_2_O_2_, hydrogen peroxide; HESC, human endometrial stromal cells; IFN-γ, interferon gamma; IL, interleukin; i.p., intraperitoneal; IVM, in vitro maturation; MDA, malondialdehyde; MII, metaphase II; MMP, matrix metalloproteinase; MTT, methylthiazolyldiphenyl-tetrazolium bromide; NAC, N-acetylcysteine; NF-κB, nuclear factor kappa B; pERK/ERK, phosphorylated extracellular signal-regulated kinase/total extracellular signal-regulated kinase; PR-A, progesterone receptor A; PR-B, progesterone receptor B; ROS, reactive oxygen species; SCID, severe combined immunodeficiency; SLC1A5, solute carrier family 1 member 5; SOD, superoxide dismutase; TAC, total antioxidant capacity; TNF-α, tumor necrosis factor alpha; VAS, visual analogue scale; VCAM1, vascular cell adhesion molecule 1.

**Table 2:** Pain outcomes in clinical and translational studies of NAC in endometriosis.

Study	Design	NAC Regimen	Pain Measure	Baseline	Post-Treatment	*p*-Value	Notes/Caveats
Porpora et al., 2013 [46]	Observational cohort	600 mg three times daily, 3 days/week, 3 months	VAS dysmenorrhea, dyspareunia, chronic pelvic pain	6.43 ± 3.39; 2.83 ± 3.19; 2.47 ± 3.82	3.11 ± 3.33; 1.38 ± 2.62; 0.77 ± 2.09	*p* = 0.001; *p* = 0.027; *p* = 0.015	24/47 cancelled surgery vs. 1/45 controls.
Ray et al., 2015 [49]	Human PF/LDL model with mouse and rat assays	1 mM during LDL oxidation; no systemic NAC dosing	Hargreaves paw-withdrawal latency (rats)	Oxidized lipoproteins induced nociception	NAC suppressed oxidized-lipoprotein-induced nociception	*p* = 0.037 for NAC × time interaction	Mechanistic pain model; mouse assay assessed thermoregulation.
Lete et al., 2018 [52]	Open-label non-comparative study	Daily dose delivered as 2 tablets/day for 6 months (NAC 600 mg + alpha-lipoic acid 200 mg + bromelain 25 mg + zinc 10 mg)	VAS pelvic pain	Mean VAS 6.68; 92.7% with VAS > 4	Mean VAS 4.55 ± 1.97 at 3 months and 3.52 ± 1.91 at 6 months; 87.2% and 82.7% with VAS > 4	*p* = 0.074 (proportion at 3 months); *p* < 0.05 (proportion at 6 months); *p* < 0.0001 for VAS change	Combination product; NSAID use declined; 52 withdrawals overall, including 13 due to adverse events.
Fadin et al., 2020 [54]	Open-label study versus historical control	150 mg/day for 2 months with quercetin 200 mg and turmeric 210 mg	NRS dysmenorrhea, pelvic pain, dyspareunia	6.1; 5.7; 5.3	2.8; 2.1; 2.5	*p* < 0.001 for all three	Combination product; historical control.
Asgari et al., 2022 [57]	Randomized postoperative trial	600 mg three times daily, 3 days/week, 3 months plus COC	VAS pelvic pain	Comparable between groups	No significant difference versus COC alone	*p* = 0.17 (3 months), *p* = 0.106 (6 months)	No placebo; participants aware of treatment allocation; global pelvic pain only.
Anastasi et al., 2023 [58]	Prospective single-cohort study	600 mg, 3 tablets/day, 3 consecutive days/week, 3 months	VAS dysmenorrhea, dyspareunia, chronic pelvic pain	6.9 ± 2.0; 6.5 ± 1.7; 7.2 ± 1.8	4.8 ± 1.8; 4.9 ± 1.7; 5.7 ± 2.0	*p* < 0.0001 for dysmenorrhea; *p* < 0.001 for dyspareunia and chronic pelvic pain	NSAID use also decreased (*p* = 0.001); no control group.

Abbreviations: COC, combined oral contraceptives; CPP, chronic pelvic pain; LDL, low-density lipoprotein; NAC, N-acetylcysteine; NRS, numerical rating scale; NSAID, nonsteroidal anti-inflammatory drug; PF, peritoneal fluid; VAS, visual analogue scale.

**Table 3:** Lesion burden, lesion activity, and endometrioma size outcomes.

Study	Design	Model	Lesion Measure	NAC-Related Condition	Comparator	*p*-Value
Ngô et al., 2009 [44]	Murine xenograft	Nude mice with human implants	Histological pathology score, 0–3	NAC: 1.19 ± 0.13	PBS: 2.0 ± 0.25	*p* < 0.025
Pittaluga et al., 2010 [45]	Experimental murine study	BALB/c mice	Endometrioma weight (mg)	29 ± 4 mg *	74 ± 9 mg *	*p* < 0.01
Porpora et al., 2013 [46]	Observational cohort	Human ovarian endometrioma	Diameter change; volume change; cysts decreased/disappeared	−1.53 ± 11.43 mm; +3.20 ± 32.7 mL; 62% decreased or disappeared	+6.62 ± 16.14 mm; +32.1 ± 89.9 mL; 28% decreased or disappeared	*p* = 0.001 (diameter); *p* = 0.012 (volume)
Onalan et al., 2014 [47]	Randomized rat study	Rat peritoneal implants	Mean implant area	30.5 ± 21.9 mm^2^ after NAC	61.2 ± 57.9 mm^2^ before NAC in the same group (within-group comparison)	*p* = 0.043
Agostinis et al., 2015 [50]	Experimental murine study (combination therapy)	SCID mouse model	Cyst number and size	Fewer and smaller cysts	Untreated	*p* < 0.001 for cyst number
Asgari et al., 2022 [57]	Randomized postoperative trial	Human recurrence after surgery	Recurrence rate at 6 months	1/48 at 3 months; 7/48 (14.6%) at 6 months	1/52 at 3 months; 10/52 (19.2%) at 6 months	*p* = 0.6
Anastasi et al., 2023 [58]	Prospective single-cohort study	Human endometrioma	Mean diameter	36.5 ± 25.4 mm to 33.0 ± 23.5 mm	No control	*p* < 0.001
Ma et al., 2025 [61]	Rat ferroptosis model	Rat endometriosis model	Lesion volume (mm^3^); Haber adhesion score	Erastin + NAC: lesion volume 34.9 ± 2.6 mm^3^; adhesion score 4.3 ± 0.4	Erastin: lesion volume 21.2 ± 2.0 mm^3^; adhesion score 2.7 ± 0.2	*p* < 0.05 vs. Erastin

Abbreviations: COC, combined oral contraceptives; SCID, severe combined immunodeficiency; mm, millimeters; mL, milliliters; mg, milligrams. In the study by Onalan et al. [47], the NAC result reflects a within-group pre/post comparison. Agostinis et al. [50] evaluated a combination of NAC, alpha-lipoic acid, and bromelain. * Pittaluga et al. [45] report lesion weights as 29 ± 4 g and 74 ± 9 g in the running text, although these values exceed the reported body weight of the animals (22.5 ± 0.7 g). In the original publication, the figure legend and *y*-axis of Figure 1A consistently use milligrams, indicating that the text values reflect a typographical error.

**Table 4:** Fertility-related outcomes.

Study	Design	NAC Regimen	Outcome Measure	Result	*p*-Value/Caveat
Porpora et al., 2013 [46]	Observational cohort	600 mg three times daily, 3 days/week	Pregnancies during observation	8 pregnancies in the NAC group versus 6 in untreated controls	Not reported
Giorgi et al., 2016 [51]	In vitro translational study	1.5 mM in IVM medium	Normal MII oocytes	62.22% with NAC vs. 51.35% with endometriosis follicular fluid alone	*p* < 0.01
Giorgi et al., 2021 [56]	In vitro translational study	1.5 mM in culture medium	Embryo hatching rate	44.44% with NAC vs. 12.5% with endometriosis follicular fluid alone	*p* = 0.020
Anastasi et al., 2023 [58]	Prospective single-cohort study	600 mg, 3 tablets/day, 3 days/week	Pregnancy within 6 months among women with reproductive desire	39 spontaneous pregnancies plus 6 pregnancies through ART among 52 women	Descriptive finding; no comparator
Heshmati et al., 2026 [62]	Double-blind placebo-controlled randomized trial	1200 mg/day orally, 6-week intervention	Oocyte/embryo quality and pregnancy	Lower defunct oocytes (4.9% vs. 6.1%), superior embryo quality numerically; 3 pregnancies and 1 live birth reported to date	Not statistically significant for oocyte/embryo outcomes

Abbreviations: ART, assisted reproductive technology; IVM, in vitro maturation; MII, metaphase II; NAC, N-acetylcysteine.

**Table 5:** Inflammatory, oxidative stress, and molecular markers.

Study	Model	Marker(s)	NAC Effect	Statistical Significance
Ngô et al., 2009 [44]	Primary cells	H_2_O_2_, GSH, SOD/catalase, pERK/ERK	Reduced H_2_O_2_ and proliferation; normalized pERK/ERK	Dose-dependent; assay-specific
Pittaluga et al., 2010 [45]	Murine	COX-2	Protein and gene expression reduced	*p* < 0.01
Pittaluga et al., 2010 [45]	Murine	MMP-9	Activity decreased by >60%	*p* < 0.01
Pittaluga et al., 2010 [45]	Murine	Ki-67	Labeling index reduced by 54%	*p* < 0.01
Pittaluga et al., 2010 [45]	Murine	E-cadherin	Relocation to cell–cell borders increased by 67%	Not specified
Onalan et al., 2014 [47]	Rat	Serum TNF-α	41.7 to 17.25 pg/mL	*p* = 0.01
Onalan et al., 2014 [47]	Rat	Peritoneal TNF-α	37.5 to 19.2 pg/mL	*p* = 0.06
Agostinis et al., 2015 [50]	In vitro endothelial model	VCAM1	Prevented TNF-α-induced upregulation (only in combination)	*p* < 0.05 for NAC/alpha-lipoic acid/bromelain combination; individual compounds not significant
Lu et al., 2020 [53]	Rat	ROS levels	Significantly decreased	*p* < 0.05
Lu et al., 2020 [53]	Rat	LC3 and Beclin-1	Downregulated	*p* < 0.05
Fadin et al., 2020 [54]	Inflamed endometrium model	IL-6 and TNF-α	Decreased with increasing formula concentration	IL-6: dose-dependent reduction; TNF-α: non-monotonic (rebound at 380 µg/mL)
Woo et al., 2020 [55]	12Z cells	Migration; MMP-2/-9	Suppressed iron-induced migration and MMP-2/-9 expression	*p* < 0.05 (iron-induced migration, MMP-2/-9 expression)
Anastasi et al., 2023 [58]	Human	CA125	45.55 to 35.6 U/mL	*p* = 0.001
Karakoç, 2023 [59]	12Z and HESC cells	β-tubulin and GRP78	β-tubulin decreased and GRP78 increased in 12Z cells; neither change was significant in HESC cells	*p* = 0.0339 and *p* = 0.0394
Karakoç et al., 2025 [60]	12Z and HESC cells	ER stress and metabolomics	Induced ER stress and metabolic alterations, especially with IFN-γ	Endpoint-dependent; *p* ≤ 0.001 to *p* < 0.05
Ma et al., 2025 [61]	hEM15A + rat	ROS/c-Myc/SLC1A5, Gln/Glu, GSH, Fe^2+^, MDA, GPX4, serum TNF-α, IL-6, IL-1β	Reversed several Erastin-induced redox and ferroptosis-related changes; reduced serum TNF-α, IL-6, and IL-1β versus Erastin alone	Endpoint-dependent; cytokines *p* < 0.001 *
Heshmati et al., 2026 [62]	Human	TAC	Significantly increased	*p* = 0.031
Heshmati et al., 2026 [62]	Human	SOD	Increased, not significant	*p* = 0.467
Heshmati et al., 2026 [62]	Human	Bcl-2/Bax/Caspase-3	Bcl-2 increased; Bax and Caspase-3 decreased, all non-significant	Not statistically significant
Coelho et al., 2026 [63]	12Z/Ishikawa 2D/3D	ROS, GSH/GSSG, total GSH, lipid/protein/DNA damage	Restored viability; reduced lipid and protein oxidation; DNA damage persisted	Endpoint-dependent

* In the original publication [61], the Results section and Figure 6 report higher serum TNF-α, IL-6, and IL-1β levels with Erastin and significant reductions after NAC co-treatment, whereas the abstract states that Erastin reduced these inflammatory markers and that this effect was mitigated by NAC. Abbreviations: Bax, BCL2-associated X protein; Bcl-2, B-cell lymphoma 2; COX-2, cyclooxygenase-2; ER, endoplasmic reticulum; GRP78, glucose-regulated protein 78; LC3, microtubule-associated protein 1 light chain 3; MMP, matrix metalloproteinase; NF-κB, nuclear factor kappa B; ROS, reactive oxygen species; SOD, superoxide dismutase; TAC, total antioxidant capacity; VCAM1, vascular cell adhesion molecule 1; Fe^2+^, ferrous iron; GPX4, glutathione peroxidase 4; GSH, glutathione; GSH/GSSG, reduced glutathione/glutathione disulfide ratio; H_2_O_2_, hydrogen peroxide; IL, interleukin; MDA, malondialdehyde; pERK/ERK, phosphorylated extracellular signal-regulated kinase/total extracellular signal-regulated kinase; SLC1A5, solute carrier family 1 member 5; Gln, glutamine; Glu, glutamate; 2D, two-dimensional; 3D, three-dimensional.

**Table 6:** Cellular proliferation, viability, migration, invasion, and stress-response outcomes.

Study	Model	Endpoint	NAC Effect	Magnitude
Foyouzi et al., 2004 [42]	Endometrial stromal cells	Proliferation (thymidine incorporation and MTT)	Dose-dependent inhibition	52–85% at 10–30 mM (thymidine); 95% at 30 mM (MTT)
Wu and Guo, 2006 [43]	YHES cell line	Proliferation (MTT)	Suppression	35–50% at 10–30 mM; 63% suppression of H_2_O_2_-induced proliferation
Ngô et al., 2009 [44]	Primary endometriotic cells	Proliferation; pERK/ERK	Reduced H_2_O_2_-linked proliferation and ERK activation	NAC 6.4 mM reduced H_2_O_2_ and proliferation; 10 mM normalized pERK/ERK
Matsuzaki and Darcha, 2014 [48]	Patient-derived endometrial/endometriotic stromal cells	Proliferation; migration/invasion; fibrosis-related endpoints	Reduced proliferation; no significant effect on migration/invasion or contraction; αSMA mRNA decreased only in endometrial cells	5–10 mM; exact values not reported
Woo et al., 2020 [55]	12Z cells	Iron-induced migration	Suppressed	Reduced iron-stimulated migration with associated suppression of MMP-2/-9; magnitude not numerically specified
Karakoç, 2023 [59]	12Z and HESC cells	Proliferation and migration	Reduced proliferation and migration in 12Z cells	IC50 3.87 × 10^−9^ M; reduced wound confluence at 48 h; β-tubulin ↓; GRP78 ↑; ER tracker ↑
Karakoç et al., 2025 [60]	12Z and HESC cells	Proliferation and organelle stress	Reduced 12Z cell proliferation; altered ER and mitochondrial function	Antiproliferative effect enhanced with IFN-γ; other effects were endpoint-dependent
Ma et al., 2025 [61]	hEM15A cells	Ferroptosis sensitivity	Reversed Erastin-induced ferroptosis-related changes	ROS/c-Myc/SLC1A5, Fe^2+^, MDA decreased; GSH, GPX4 increased
Coelho et al., 2026 [63]	12Z/Ishikawa 2D/3D	Viability; biomolecular damage	Restored viability and reduced lipid/protein oxidation; DNA damage persisted	5 mM in 2D; 1.25 mM in 3D; endpoint-dependent

Abbreviations: EGCG, epigallocatechin-3-gallate; EMT, epithelial–mesenchymal transition; ER, endoplasmic reticulum; GRP78, glucose-regulated protein 78; H_2_O_2_, hydrogen peroxide; HESC, human endometrial stromal cells; MMP, matrix metalloproteinase; MTT, methylthiazolyldiphenyl-tetrazolium bromide; NAC, N-acetylcysteine; ROS, reactive oxygen species.

## Data Availability

No new data were created or analyzed in this study. Data sharing is not applicable to this article.

## Supplementary Materials

The following supporting information can be downloaded at: https://www.mdpi.com/article/10.3390/antiox15070880/s1: Table S1: PRISMA 2020 Checklist; Table S2: Domains used for structured appraisal of mechanistic in vitro and ex vivo evidence; Table S3: Criteria used for overall appraisal.

## Abbreviations

The following abbreviations are used in this manuscript: 2D—two-dimensional; 3D—three-dimensional; APA—American Psychological Association; AR—androgen receptor; ART—assisted reproductive technology; Bax—BCL2-associated X protein; Bcl-2—B-cell lymphoma 2; CA125—cancer antigen 125; CINAHL—Cumulative Index to Nursing and Allied Health Literature; COC—combined oral contraceptives; COX-2—cyclooxygenase-2; CPP—chronic pelvic pain; EGCG—epigallocatechin-3-gallate; EMT—epithelial–mesenchymal transition; ER—endoplasmic reticulum; ERK—extracellular signal-regulated kinase; FasL—Fas ligand; Gln—glutamine; Glu—glutamate; GPX4—glutathione peroxidase 4; GRADE—Grading of Recommendations Assessment, Development and Evaluation; GRP78—glucose-regulated protein 78; GSH—glutathione; GSH/GSSG—reduced glutathione/glutathione disulfide ratio; HESC—human endometrial stromal cells; H2O2—hydrogen peroxide; IC50—half-maximal inhibitory concentration; IFN-γ—interferon gamma; IL—interleukin; IVM—in vitro maturation; LC3—microtubule-associated protein 1 light chain 3; MDA—malondialdehyde; MII—metaphase II; MMP—matrix metalloproteinase; MRI—magnetic resonance imaging; MTT—methylthiazolyldiphenyl-tetrazolium bromide; NAC—N-acetylcysteine; NF-κB—nuclear factor kappa B; NKA—neurokinin A; NK2 receptor—neurokinin 2 receptor; NRS—numerical rating scale; NSAID—nonsteroidal anti-inflammatory drug; pERK/ERK—phosphorylated extracellular signal-regulated kinase/total extracellular signal-regulated kinase; OSF—Open Science Framework; PR-A—progesterone receptor A; PR-B—progesterone receptor B; PRISMA—Preferred Reporting Items for Systematic Reviews and Meta-Analyses; rASRM—revised American Society for Reproductive Medicine; RoB 2—Risk of Bias 2 tool; ROBINS-I—Risk Of Bias In Non-randomized Studies of Interventions; ROS—reactive oxygen species; SCID—severe combined immunodeficiency; SOD—superoxide dismutase; SYRCLE—Systematic Review Centre for Laboratory Animal Experimentation; TAC—total antioxidant capacity; TBARS—thiobarbituric acid reactive substances; TGF-β—transforming growth factor beta; TNF-α—tumor necrosis factor alpha; VAS—visual analogue scale; VCAM1—vascular cell adhesion molecule 1.

## Appendix A

**Table A1:** Risk of bias and quality assessment of included studies.

Study	Design/Model	Tool	Overall Judgment	Main Reason
Foyouzi et al., 2004 [42]	In vitro stromal-cell study	Structured in vitro appraisal	Some concerns	Objective proliferation endpoints and dose–response design, but no masking and limited broader reproducibility reporting.
Wu and Guo, 2006 [43]	In vitro cell-line study	Structured in vitro appraisal	Some concerns	Clear comparative pharmacologic design, but no blinding and limited reporting beyond core assays.
Ngô et al., 2009 [44]	Patient-derived ex vivo/in vitro study plus murine xenograft	SYRCLE + structured in vitro appraisal	Some concerns/unclear	Patient-derived cells and blinded histology; allocation and housing unclear; millimolar NAC.
Pittaluga et al., 2010 [45]	Murine in vivo study	SYRCLE	Some concerns/unclear	Strong biological effect, but reporting of allocation, concealment, and blinding was limited.
Porpora et al., 2013 [46]	Observational clinical cohort	ROBINS-I	Serious	Treatment depended on acceptance versus refusal of NAC, creating substantial confounding and selection bias.
Onalan et al., 2014 [47]	Randomized rat study	SYRCLE	Some concerns	Group allocation and blinded measurements were described, but several SYRCLE domains were insufficiently reported.
Matsuzaki and Darcha, 2014 [48]	Patient-derived in vitro study (NAC part)	Structured in vitro appraisal	Some concerns	Patient-derived cells and multiple assays, but millimolar NAC exposure and no masking information.
Ray et al., 2015 [49]	Hybrid mechanistic/translational study	SYRCLE	Some concerns	Rat Hargreaves assay randomized/blinded; mouse thermoregulation separate; allocation and housing details incompletely reported.
Agostinis et al., 2015 [50]	Hybrid in vivo/in vitro study	SYRCLE	Some concerns/unclear	Clear experimental effects, but incomplete reporting of randomization and masking procedures.
Giorgi et al., 2016 [51]	Translational in vitro oocyte model	Structured in vitro appraisal	Some concerns	Biologically relevant translational model, but indirect with respect to clinical exposure.
Lete et al., 2018 [52]	Open-label uncontrolled clinical study	ROBINS-I	Critical	Multi-ingredient regimen, no comparator, patient-reported outcomes, and substantial attrition.
Lu et al., 2020 [53]	Rat in vivo study	SYRCLE	Some concerns/unclear	Biomarker endpoints were objective, but reporting of randomization, concealment, and blinding was limited.
Fadin et al., 2020 [54]	Open-label historical-control study	ROBINS-I	Critical	Small study with historical control, expectation effects, and uncontrolled co-intervention.
Woo et al., 2020 [55]	In vitro plus BALB/c murine study	SYRCLE + structured in vitro appraisal	Some concerns/unclear	Mechanistically informative; NAC tested in vitro only; mouse experiment (*n* = 3/group) tests iron, not NAC; SYRCLE domains mostly insufficiently reported.
Giorgi et al., 2021 [56]	Translational in vitro embryo model	Structured in vitro appraisal	Some concerns	Added translational value, but remained an ex vivo surrogate model.
Asgari et al., 2022 [57]	Randomized postoperative clinical trial	RoB 2	Some concerns	Randomization described, but no placebo, participant awareness of treatment allocation, and subjective pain outcome.
Anastasi et al., 2023 [58]	Prospective single-cohort clinical study	ROBINS-I	Critical	No comparator and reliance on within-group change for subjective and structural outcomes.
Karakoç, 2023 [59]	In vitro mechanistic study	Structured in vitro appraisal	Some concerns	Informative dose-finding and mechanistic readouts, but no masking and unusually low IC50 range requiring caution.
Karakoç et al., 2025 [60]	In vitro mechanistic study	Structured in vitro appraisal	Some concerns	Cytokine-context experiments were mechanistically rich, but remained model-dependent and unblinded.
Ma et al., 2025 [61]	hEM15A cell study plus rat model	SYRCLE + structured in vitro appraisal	Some concerns/unclear	Pathway-directed design; small animal groups; allocation and blinding unclear; NAC used as Erastin antagonist.
Heshmati et al., 2026 [62]	Double-blind placebo-controlled RCT	RoB 2	Some concerns	Methodologically stronger, but small sample, post-randomization exclusions, and mostly non-significant downstream endpoints.
Coelho et al., 2026 [63]	2D/3D in vitro cell-culture study	Structured in vitro appraisal	Some concerns	2D/3D design with biochemical endpoints; immortalized cells, H_2_O_2_ challenge, millimolar NAC, no masking.

Abbreviations: NAC, N-acetylcysteine; RoB 2, revised Cochrane risk-of-bias tool for randomized trials; ROBINS-I, Risk Of Bias In Non-randomized Studies of Interventions; SYRCLE, Systematic Review Centre for Laboratory Animal Experimentation risk-of-bias tool.
