# Supplementary material for: N-Acetylcysteine in Endometriosis: A Systematic Review of Biological Rationale and Clinical Evidence
Source: Antioxidants (Basel). 2026 Jul 16;15(7):880. doi: 10.3390/antiox15070880 (PMC13405467; doi:10.3390/antiox15070880)
Supplement: Supplementary file 1 [file antioxidants-15-00880-s001.zip › Supplementary Table S3.pdf]

Supplementary Table S3. Criteria used for overall appraisal.

**Overall appraisal**	**Explanation**
Low concern	The model was relevant to the question, NAC exposure was clearly reported, controls were appropriate, endpoints were objective and mechanism-linked, and replication and statistical reporting were adequate. No major translational limitation dominated interpretation.
Some concerns	The study was mechanistically informative, but one or more domains limited interpretation, such as use of an indirect or immortalized model, supraphysiological NAC concentrations, limited masking, incomplete replication details, restricted endpoint scope, or incomplete translational relevance.
High concern	Several domains substantially weakened mechanistic interpretation, such as poorly characterized models, unclear NAC exposure, weak or nonspecific endpoints, inadequate controls, major reporting gaps, or conclusions extending beyond the experimental design.
Unclear	Reporting was insufficient to appraise one or more major domains, including cell source, controls, replication, masking, dose, exposure duration, or statistical handling.
Hybrid studies	For studies combining in vivo and cell-based or ex vivo components, murine and rat in vivo components were assessed with SYRCLE, while cell-based or ex vivo components were assessed with the structured appraisal described in Supplementary Table S1.
