# Supplementary material for: N-Acetylcysteine in Endometriosis: A Systematic Review of Biological Rationale and Clinical Evidence
Source: Antioxidants (Basel). 2026 Jul 16;15(7):880. doi: 10.3390/antiox15070880 (PMC13405467; doi:10.3390/antiox15070880)
Supplement: Supplementary file 1 [file antioxidants-15-00880-s001.zip › Supplementary Table S1. PRISMA_2020_checklist.pdf]

PRISMA 2020 Checklist

Section and Topic	Item #	Checklist item	Location where item is reported
**TITLE**			
Title	1	Identify the report as a systematic review.	Title: "N-Acetylcysteine in Endometriosis: A Systematic Review of Biological Rationale and Clinical Evidence".
**ABSTRACT**			
Abstract	2	See the PRISMA 2020 for Abstracts checklist.	Structured abstract (Background, Methods, Results, Conclusions) and Keywords. See also the PRISMA 2020 for Abstracts checklist, if submitted separately.
**INTRODUCTION**			
Rationale	3	Describe the rationale for the review in the context of existing knowledge.	Introduction, paragraphs 1–5: disease burden, diagnostic and therapeutic limitations, inflammatory-redox rationale, and biological plausibility of NAC as a non-hormonal candidate.
Objectives	4	Provide an explicit statement of the objective(s) or question(s) the review addresses.	End of Introduction: primary aim (to evaluate the available evidence on NAC across clinical and preclinical settings) and secondary aim (to determine which biological pathways and disease dimensions are most consistently implicated).
**METHODS**			
Eligibility criteria	5	Specify the inclusion and exclusion criteria for the review and how studies were grouped for the syntheses.	Section 2.2 Eligibility criteria and Section 2.7 Data synthesis. Eligible studies included human clinical studies, murine and rat in vivo studies, bovine reproductive models, patient-derived ex vivo systems, and mechanistic in vitro studies evaluating NAC in endometriosis-related settings. NAC-only and NAC-containing combination regimens were interpreted separately.
Information sources	6	Specify all databases, registers, websites, organisations, reference lists and other sources searched or consulted to identify studies. Specify the date when each source was last searched or consulted.	Section 2.3 Information sources and search strategy: PubMed/MEDLINE; EBSCOhost (Academic Search Premier, APA PsycArticles, APA PsycInfo, CINAHL, MEDLINE); Bielefeld Academic Search Engine (BASE); and manual reference-list screening. Searches covered database inception to 24 May 2026.
Search strategy	7	Present the full search strategies for all databases, registers and websites, including any filters and limits used.	Section 2.3 reports the full PubMed/MEDLINE and EBSCOhost free-text search string: "(endometriosis OR endometriotic OR endometrioma OR adenomyosis) AND (n-acetylcysteine OR n-acetyl cysteine OR NAC OR acetylcysteine OR acetyl-cysteine OR N-acetyl-L-cysteine OR N acetyl L cysteine)". The BASE all-fields query was "(n-acetylcysteine OR n-acetyl cysteine) AND endometriosis". No date, language, or species filters were applied.
Selection process	8	Specify the methods used to decide whether a study met the inclusion criteria of the review, including how many reviewers screened each record and each report retrieved, whether they worked independently, and if applicable, details of automation tools used in the process.	Section 2.4 Study selection: two reviewers (R.W., S.K.) independently screened titles/abstracts and full-text reports; disagreements were resolved by discussion with A.Y. and S.G.V.; no automation tools were used for study selection or data extraction.
Data collection process	9	Specify the methods used to collect data from reports, including how many reviewers collected data from each report, whether they worked independently, any processes for obtaining or confirming data from study investigators, and if applicable, details of automation tools used in the process.	Section 2.5 Data extraction: data were extracted directly from full texts into a structured evidence table; extracted variables are listed; combination regimens were recorded separately from NAC-only interventions. Section 2.4 states that no automation tools were used for data extraction.
Data items	10a	List and define all outcomes for which data were sought. Specify whether all results that were compatible with each outcome domain in each study were sought (e.g. for all measures, time points, analyses), and if not, the methods used to decide which results to collect.	Sections 2.2 and 2.5. Outcome domains included pain, endometrioma or lesion burden, postoperative recurrence, fertility-related outcomes, oxidative-stress and inflammatory signaling, migration/invasion and matrix remodeling, endoplasmic-reticulum stress, autophagy-related readouts, ferroptosis-related mechanisms, and fibrosis-associated pathways. Compatible results per study were extracted where reported.
	10b	List and define all other variables for which data were sought (e.g. participant and intervention characteristics, funding sources). Describe any	Section 2.5: first author, year, journal, study design, country where applicable, population/model, sample size, diagnostic or modelling approach, NAC regimen (dose, route, duration, schedule), comparator/control, follow-up, outcome definitions, and main

Section and Topic	Item #	Checklist item	Location where item is reported
		assumptions made about any missing or unclear information.	quantitative and qualitative findings. Missing or unclear items are reported as such in Tables 1–6 and Appendix Table A1.
Study risk of bias assessment	11	Specify the methods used to assess risk of bias in the included studies, including details of the tool(s) used, how many reviewers assessed each study and whether they worked independently, and if applicable, details of automation tools used in the process.	Section 2.6 Risk-of-bias assessment: RoB 2 for randomized clinical trials [40]; ROBINS-I for non-randomized clinical intervention studies [41]; SYRCLE for murine and rat in vivo studies [42]; structured appraisal for mechanistic in vitro and ex vivo components. The structured appraisal domains and criteria are provided in Supplementary Tables S2 and S3. Detailed study-level judgments are presented in Appendix Table A1. No automation tools were used.
Effect measures	12	Specify for each outcome the effect measure(s) (e.g. risk ratio, mean difference) used in the synthesis or presentation of results.	Sections 2.7 and 3.4: meta-analysis was not performed because of heterogeneity. Results are presented as originally reported, including means ± SD, percentages, p-values, VAS/NRS scores, lesion measures, biomarker changes, and qualitative effect directions in Tables 2–6.
Synthesis methods	13a	Describe the processes used to decide which studies were eligible for each synthesis (e.g. tabulating the study intervention characteristics and comparing against the planned groups for each synthesis (item #5)).	Sections 2.7 and 3.4: studies were assigned to synthesis categories according to reported outcomes (pain, lesion burden/endometrioma size, fertility-related outcomes, inflammatory/oxidative/molecular markers, cellular proliferation/viability/migration/invasion, and tolerability). NAC-only interventions and NAC-containing combination regimens were interpreted separately.
	13b	Describe any methods required to prepare the data for presentation or synthesis, such as handling of missing summary statistics, or data conversions.	Section 2.7: no data conversions or imputations were performed for synthesis. Missing or unclear quantitative data are indicated in the evidence tables.
	13c	Describe any methods used to tabulate or visually display results of individual studies and syntheses.	Section 3.4: results are tabulated in Tables 2–6 by outcome category; Table 1 summarizes study characteristics; Appendix Table A1 summarizes risk-of-bias/quality judgments; Figure 1 illustrates mechanistic correspondence; Figure 2 presents the PRISMA 2020 flow diagram.
	13d	Describe any methods used to synthesize results and provide a rationale for the choice(s). If meta-analysis was performed, describe the model(s), method(s) to identify the presence and extent of statistical heterogeneity, and software package(s) used.	Sections 2.7, 3.4, and 3.5: narrative synthesis was used because of marked heterogeneity in study design, populations, model systems, NAC regimens, comparators, follow-up duration, and outcome definitions. No meta-analysis was conducted and no statistical software was used for pooling.
	13e	Describe any methods used to explore possible causes of heterogeneity among study results (e.g. subgroup analysis, meta-regression).	Sections 3.4, 3.5, and 4: heterogeneity was explored qualitatively by clinical setting, NAC regimen (monotherapy versus combination), disease context, study design, model system, and endpoint category.
	13f	Describe any sensitivity analyses conducted to assess robustness of the synthesized results.	Not applicable. Quantitative synthesis was not performed; sensitivity analyses were therefore not conducted (Section 2.7).
Reporting bias assessment	14	Describe any methods used to assess risk of bias due to missing results in a synthesis (arising from reporting biases).	Not formally assessed with a reporting-bias tool given the absence of meta-analysis. Publication, selective-reporting, and evidence-sparsity considerations are discussed narratively in Sections 3.3 and 4.
Certainty assessment	15	Describe any methods used to assess certainty (or confidence) in the body of evidence for an outcome.	Certainty of evidence was not graded with GRADE. Overall confidence is discussed narratively in Section 4 and reflected in design-specific risk-of-bias/quality judgments in Section 3.3 and Appendix Table A1; this is acknowledged as a limitation.
**RESULTS**			
Study selection	16a	Describe the results of the search and selection process, from the number of records identified in the search to the number of studies included in the review, ideally using a flow diagram.	Section 3.1 Study selection and Figure 2: PubMed/MEDLINE retrieved 47 records, EBSCOhost 85, and BASE 79 (211 records total). After duplicate removal (n = 116), 95 records were screened; 71 were excluded by title/abstract; 24 full-text reports were assessed; 2 were excluded; 22 studies were included in the final synthesis.
	16b	Cite studies that might appear to meet the inclusion criteria, but which were excluded, and explain why they were excluded.	Section 3.1: two full-text reports were excluded because they did not meet the inclusion criteria. Reference-list screening did not identify additional eligible studies.
Study characteristics	17	Cite each included study and present its characteristics.	Section 3.2 and Table 1: characteristics of all 22 included studies are presented.

PRISMA

PRISMA 2020 Checklist

Section and Topic	Item #	Checklist item	Location where item is reported
Risk of bias in studies	18	Present assessments of risk of bias for each included study.	Section 3.3 Risk of Bias across Studies; detailed per-study judgments are presented in Appendix Table A1.
Results of individual studies	19	For all outcomes, present, for each study: (a) summary statistics for each group (where appropriate) and (b) an effect estimate and its precision (e.g. confidence/credible interval), ideally using structured tables or plots.	Tables 2–6 present per-study results by outcome category, including pain, lesion burden/endometrioma size, fertility-related outcomes, inflammatory/oxidative/molecular markers, cellular proliferation/viability/migration/invasion, and tolerability, with baseline/post-treatment values, comparator values, effect direction, magnitude, and p-values where reported.
Results of syntheses	20a	For each synthesis, briefly summarise the characteristics and risk of bias among contributing studies.	Section 3.4 and Section 3.5 summarize the characteristics, design mix, and risk-of-bias considerations among studies contributing to each outcome category.
	20b	Present results of all statistical syntheses conducted. If meta-analysis was done, present for each the summary estimate and its precision (e.g. confidence/credible interval) and measures of statistical heterogeneity. If comparing groups, describe the direction of the effect.	Section 3.4 sub-sections and Tables 2–6. No pooled statistical synthesis was performed; direction and magnitude of effect are summarized per study.
	20c	Present results of all investigations of possible causes of heterogeneity among study results.	Discussion (Section 4): heterogeneity is discussed qualitatively by clinical setting, intervention context, regimen, model system, and endpoint category.
	20d	Present results of all sensitivity analyses conducted to assess the robustness of the synthesized results.	Not applicable. No meta-analysis was conducted; sensitivity analyses were therefore not performed (Section 2.7).
Reporting biases	21	Present assessments of risk of bias due to missing results (arising from reporting biases) for each synthesis assessed.	Not formally assessed. Considerations regarding selective reporting, publication bias, and sparse evidence are addressed narratively in Sections 3.3 and 4 and acknowledged as limitations.
Certainty of evidence	22	Present assessments of certainty (or confidence) in the body of evidence for each outcome assessed.	Certainty of evidence was not graded with GRADE. Qualitative confidence judgments are provided in Section 4 and in Section 3.3 / Appendix Table A1; this is acknowledged as a limitation of the review.
**DISCUSSION**			
Discussion	23a	Provide a general interpretation of the results in the context of other evidence.	Discussion (Section 4): interpretation of clinical, murine, rat, bovine, ex vivo, and in vitro findings in the context of endometriosis pathobiology and NAC pharmacology.
	23b	Discuss any limitations of the evidence included in the review.	Section 4: limitations of the included evidence, including few clinical studies, only two randomized trials, short follow-up, inconsistent outcome definitions, supraphysiological in vitro concentrations, combination regimens, absence of concurrent controls in several clinical studies, and model-dependent preclinical evidence.
	23c	Discuss any limitations of the review processes used.	Section 4: limitations of the review process, including narrative-only synthesis because of heterogeneity, no GRADE rating, and no formal reporting-bias assessment.
	23d	Discuss implications of the results for practice, policy, and future research.	Section 4 and Section 5 Conclusions: implications for practice and research, including the absence of a standard-of-care recommendation, the need for placebo-controlled and phenotype-stratified NAC monotherapy trials, separate pain-domain assessment, standardized endometrioma measurement, and pharmacodynamic oxidative-stress markers.
**OTHER INFORMATION**			
Registration and protocol	24a	Provide registration information for the review, including register name and registration number, or state that the review was not registered.	Section 2.1: prospectively registered on the Open Science Framework (OSF); registration DOI https://doi.org/10.17605/OSF.IO/CHKBQ.
	24b	Indicate where the review protocol can be accessed, or state that a protocol was not prepared.	Section 2.1: protocol accessible via the OSF registration above (https://doi.org/10.17605/OSF.IO/CHKBQ).
	24c	Describe and explain any amendments to information provided at	Section 2.3 reports the final search strategy and final search date. During revision, the search strategy was refined by expanding NAC synonyms and searching PubMed/MEDLINE,

PRISMA 2020 Checklist

Section and Topic	Item #	Checklist item	Location where item is reported
		registration or in the protocol.	EBSCOhost, and BASE through 24 May 2026. Eligibility criteria, outcome domains, and narrative synthesis plan were unchanged.
Support	25	Describe sources of financial or non-financial support for the review, and the role of the funders or sponsors in the review.	Funding statement: "This research received no external funding." No role of funders.
Competing interests	26	Declare any competing interests of review authors.	Conflicts of Interest statement: "The authors declare no conflicts of interest."
Availability of data, code and other materials	27	Report which of the following are publicly available and where they can be found: template data collection forms; data extracted from included studies; data used for all analyses; analytic code; any other materials used in the review.	Supplementary Materials: completed PRISMA 2020 Checklist provided as Supplementary Table S1; structured appraisal domains and overall appraisal criteria provided as Supplementary Tables S2 and S3. Extracted data are presented in Tables 1–6 and Appendix Table A1. No custom analytic code was generated.

From: Page MJ, McKenzie JE, Bossuyt PM, Boutron I, Hoffmann TC, Mulrow CD, et al. The PRISMA 2020 statement: an updated guideline for reporting systematic reviews. BMJ 2021;372:n71. doi: 10.1136/bmj.n71.
This work is licensed under CC BY 4.0. To view a copy of this license, visit https://creativecommons.org/licenses/by/4.0/
