# Supplementary material for: N-Acetylcysteine in Endometriosis: A Systematic Review of Biological Rationale and Clinical Evidence
Source: Antioxidants (Basel). 2026 Jul 16;15(7):880. doi: 10.3390/antiox15070880 (PMC13405467; doi:10.3390/antiox15070880)
Supplement: Supplementary file 1 [file antioxidants-15-00880-s001.zip › Supplementary Table S2.pdf]

Supplementary Table S2. Domains used for structured appraisal of mechanistic *in vitro* and *ex vivo* evidence.

**Domain**	**Appraisal question**	**Examples of relevant criteria**
Model relevance and characterization	Is the experimental system biologically relevant to endometriosis and sufficiently described?	Primary human endometriotic or endometrial cells, patient-derived material, validated cell lines, bovine reproductive models, 2D/3D culture systems, species and tissue source, cell-line authentication where reported.
Pathological context	Does the model reproduce a disease-relevant stimulus or microenvironmental feature?	Oxidative challenge, iron overload, inflammatory cytokine exposure, endometriosis-associated follicular fluid, peritoneal-fluid components, ferroptosis induction, migration or invasion context.
NAC intervention and translational plausibility	Are NAC concentration, exposure time, route or culture conditions clearly reported and biologically interpretable?	Concentration, route or culture exposure, duration, schedule, dose-response design, co-treatment, physiological plausibility, supraphysiological or difficult-to-translate concentrations.
Endpoint and pathway specificity	Do the measured endpoints match the proposed mechanism?	Proliferation, viability, apoptosis, migration, invasion, ROS, GSH/GSSG, TAC, SOD, MMPs, ERK1/2, NF-κB, ER stress, autophagy, ferroptosis markers, oocyte/embryo endpoints.
Experimental rigor and reporting transparency	Does the design support reliable interpretation of the reported findings?	Appropriate controls, biological replication, statistical reporting, assay description, masking where reported, reproducibility details, incomplete reporting or unclear handling of variability.
